# Minocycline Attenuates Stress-Induced Behavioral Changes via Its Anti-inflammatory Effects in an Animal Model of Post-traumatic Stress Disorder

**DOI:** 10.3389/fpsyt.2018.00558

**Published:** 2018-11-06

**Authors:** Wei Wang, Rui Wang, Jingjing Xu, Xiaqing Qin, Hong Jiang, Arslan Khalid, Dexiang Liu, Fang Pan, Cyrus S. H. Ho, Roger C. M. Ho

**Affiliations:** ^1^Department of Medical Psychology and Ethics, School of Basic Medical Sciences, Shandong University, Jinan, China; ^2^Department of Psychological Medicine, Yong Loo Lin School of Medicine, National University of Singapore, Singapore, Singapore

**Keywords:** post-traumatic stress disorder, inescapable foot shock, microglia, minocycline, pro-inflammatory cytokines, NF-κB

## Abstract

Accumulating evidences have suggested that anxiety-like behavior and impairment of learning and memory are key symptoms of post-traumatic stress disorder (PTSD), and pharmacological treatment can ameliorate anxiety and cognitive impairments. Recent studies have shown that minocycline exhibits anxiolytic effects. The aims of the present study were to determine whether minocycline administration would alter anxiety-like behavior and cognitive deficits induced by inescapable foot shock (IFS) and to explore the underlying mechanisms. Male Wistar rats were exposed to the IFS protocol for a period of 6 days to induce PTSD. The PTSD-like behavior was tested using the open field test, elevated plus maze test, and Morris water maze test. The effects of minocycline on pro-inflammatory cytokines, activation of microglia, and NF-κB in the PFC and hippocampus were also examined. Treatment with minocycline significantly reversed the IFS induced behavioral and cognitive parameters (impaired learning and memory function) in stressed rats. Additionally, IFS was able to increase pro-inflammatory cytokines, activate microglia, and enhance NF-κB levels, while minocycline significantly reversed these alterations. Taken together, our results suggest that the anxiolytic effect of minocycline is related to its ability to decrease the levels of pro-inflammatory cytokines and inhibit activation of microglia and NF-κB in the PFC and hippocampus.

## Introduction

Post-traumatic stress disorder (PTSD) is one of the “trauma- and stressor-related disorders,” and its diagnostic criteria include exposure to extreme traumatic events, recurrent and intrusive traumatic memories, avoidance of traumatic event-related stimuli, cognitive impairment, negative emotions, hyper-aroused state, hyper-vigilance, clinical distress, and social impairment (1). PTSD is a global health issue and has a serious negative impact on individuals and society, the prevalence of PTSD is high and it substantially increases the risk of other psychiatric morbidities as well as medical disorders (2–4). Although the current studies have identified the molecular, neurochemical and genetic abnormalities associated with of PTSD (5), prevention and treatment of PTSD are limited (4, 6).

Based on the diagnostic criteria, stress is strongly associated with PTSD. Stress is implicated in the etiology and pathogenesis of a number of psychiatric disorders including major depressive disorder and anxiety disorders (7). Previous research has demonstrated that different types of stress have a close association with inflammatory activities in the central nervous system (CNS) (8) and peripheral circulation (9). Several studies have demonstrated that the levels of peripheral pro-inflammatory cytokines or other inflammatory markers were elevated in patients suffering from PTSD (10–12). Furthermore, studies based on the rodent models of PTSD found increased levels of cytokines in different neuroanatomical areas (13–15). These cytokines coordinate communication between neurons, microglia and astrocytes and alter the neuroendocrine and neurochemical processes, which lead to ultimate changes in behavior (16). Although, the cytokines in the CNS can travel to the peripherial circulation, the major cells that producing pro-inflammatory cytokines are microglia (17). As resident macrophages in the CNS, chronic, and severe stress can trigger the transition of microglia from a resting state to a pro-inflammatory state and produce a “cytokine storm” that can disrupt the homeostasis of the brain (18). The above studies prompted researchers to consider a new therapeutic avenue for PTSD by inhibiting the activation of microglia. Nuclear factor kappa B (NF-κB) is a transcription factor of inflammation, which translocates into the nucleus and is able to up-regulate the expression of pro-inflammatory immune response genes, including tumor necrosis factor (TNF)-α, interleukin (IL)-1β, and IL-6 (19). Inhibition of this pathway might be a potential treatment for PTSD.

It was recently reported that minocycline, the semi-synthetic tetracycline, can serve as a versatile drug for the treatment of inflammatory diseases (20), as well as psychiatric disorders (21). Accumulating evidence has shown that minocycline exhibits its potential anti-inflammatory and immune-modulatory effects in the CNS. In addition, previous studies have shown that minocycline administration is able to improve anxiety-like behavior and cognitive impairment (22–25). The beneficial effects of minocycline might partly be attributed to its ability to down-regulate pro-inflammatory cytokines (20, 21), attenuate the activation of microglia (26) and selectively inhibit the polarization of microglia toward the M1 phenotype (27). In addition, previous studies have suggested that minocycline can be a potential treatment for PTSD experienced by rodents (14). However, the cellular and molecular mechanism of minocycline remains unknown.

In the present study, rats were exposed to a 6 days inescapable foot shock (IFS) protocol followed by subsequent behavioral assessments. The levels of pro-inflammatory cytokines, activation of microglia and NF-κB in the prefrontal cortex (PFC) and hippocampus were evaluated. We hypothesized that minocycline could exert a therapeutic effect in the rat model of PTSD, and it might exhibit a neuroprotective function through anti-inflammatory effects by down-regulating the activation of microglia and NF-κB in the PFC and hippocampus. This study evaluated minocycline as a potential therapeutic agent for PTSD.

## Materials and methods

### Animals and drug treatment

Eight-weeks-old male Wistar rats weighing 190–210 g were purchased from the Animal Center of Shandong University. A total of 40 rats were housed in a controlled environment (12 h day/night cycle, 23 ± 2°C) with free access to water and food. The research procedures were approved by the Animal Ethics Committee of Shandong University.

Minocycline was dissolved in saline, and the rats were intragastrically administered with minocycline (Biottoped, China) at the dosage of 40 mg/kg/d, which were based on the methods of previous studies (25, 28). Based on the established protocol, the rats received a single dose of minocycline or vehicle 30 min before receiving daily foot shock for a total of 6 times in 6 days.

### Experimental design

Animals were randomly divided into four experimental groups: control, PTSD alone, minocycline alone and PTSD + minocycline, with 10 rats per group. After 1 week adaptation, animals were exposed to daily foot shock for 6 days (day 8–13). Then all animals were conducted with the open field (OF) test (day 14), elevated plus maze (EPM) test (day 15), Morris water maze (MWM) train (day 16–20), and test (day21) in a sequential manner. Following the behavioral tests, animals were decapitated or anesthetized for tissue preparation (day 22).

### The IFS procedure

The IFS-exposed rats received foot shocks twice a day with a break of more than 4 h in-between foot shocks, and the procedure continued for 6 days. Rats in the IFS-exposed groups were placed in a dark foot-shock box and received 15 spaced shocks (3 mA, 2 s) in 30 min with a variable shock interval. Rats in the two unexposed groups were placed into the box for 30 min without electric shock.

### Behavioral test

#### The open field (OF) test

The OF test was used to assess exploratory activities and anxiety-like behavior in an open box (29). The open field is a square wooden box with a base of 50 cm^2^ and a wall height of 50 cm, and the bottom was divided equally into 25 blocks with markers. Before each trial, the arena surface was cleaned with 75% ethanol, the rats were placed in the center of the apparatus, and the subsequent activities were recorded by a camera for 5 min. All behavioral data were counted by two independent experimenters. The evaluation of behavioral data included horizontal locomotion (the number of three limbs crossing the line), duration of time spent in the central area, rearing frequency (two forepaws lifting from the ground), and grooming frequency (licking or scratching).

#### The elevated plus maze (EPM) test

The EPM test was designed to test the animal's anxiety-like behavior by examining the duration of time spent and frequency of entries in the open arms and closed arms (30). The device is elevated 50 cm from the floor and has two open arms and two closed arms (surrounded on three sides by walls 18 cm in height) connected by a square platform. The animals were placed in the center area with their heads facing the open arm, and trajectory of the rat was recorded for 5 min with video tracking software (SMART 2.5, Spain). The device was cleaned with 75% ethanol before each trial. The percentage of time spent in the open arms and the frequency of entering the open arms were calculated.

#### The morris water maze (MWM) test

The MWM test was designed to assess learning and memory function through training and testing in a circular pool (31). The round water maze (120 cm in diameter) was divided into four quadrants, and a platform was hidden 1 cm below the surface of the water. Rats were trained to search for the platform four times a day for 5 days, and they were tested on the sixth day with the platform removed. The escape latency (time taken to find the platform) in the training day and the percentage of time spent and entry frequency in the target quadrant (the quadrant where the platform was placed) in the testing day were recorded by video tracking software (SMART 2.5, Spain).

### Biochemical determination

#### Tissue preparation

For western blotting, enzyme-linked immunosorbent assay (ELISA), and real-time PCR, rats were decapitated, and the brains were immediately removed from the rats. The PFC and hippocampus were separated on ice and frozen at −80°C. For immunohistochemistry (IHC), Rats were anesthetized with pentobarbital sodium (50 mg/kg) and transcardially perfused with 0.9% saline and then 4% paraformaldehyde dissolved in PBS. Then the brains were removed and post-fixed in 4% paraformaldehyde for 24 h at 4°C.

#### ELISA

The samples were weighed and then homogenized completely in phosphate-buffered solution (PBS). After centrifugation (1,000 × g, 10 min) of the homogenates at 4°C, supernatants were collected and stored at −80°C. The concentrations of TNF-α, IL-1β, and IL-6 were detected following the instruction of ELISA kits (Tianjin Anoric Bio-technology, Co., Ltd, China).

#### IHC

The tissues were dehydrated in a graded series of ethanol, cleared in xylene and embedded in paraffin blocks. The blocks of tissue were sectioned serially at 5 μm using a microtome. After the tissues were mounted on the slide, deparaffinated and rehydrated, they were immersed in sodium citrate for antigen retrieval using a microwave oven (medium power for 6 min, 4 times). The sections were treated with 3% H_2_O_2_ for 10 min and washed three times with PBS. These sections were blocked with 5% bovine serum albumin (BSA) in PBS for 30 min and incubated overnight with the primary antibody Iba-1 (ab5076, 1:400, Abcam) at 4°C. After three PBS washes, these sections were incubated with biotinylated-conjugated rabbit anti-goat IgG secondary antibody for 30 min at 37°C. After three PBS washes, sections were treated with SABC for 30 min at 37°C and then washed three times with PBS. A DAB kit was used for chromogenic detection, and the sections were then stained with haematoxylin and mounted. The images were captured by an OLYMPUS microscope.

#### RNA extraction and real-time PCR

Total RNA was isolated from the PFC and hippocampus of rats using RNAprep Pure Tissue Kit (Tiangen Biotech Co., Ltd, China) following the manufacturer's instructions. The RNA was quantified and analyzed for the absorbance ratios A260/280 nm using a nano-400 (Hangzhou Allsheng Instruments Co.,Ltd, China). 1 μg RNA was reverse-transcribed into first-strand cDNA using the FastKing RT kit (Tiangen Biotech Co., Ltd, China) with random primer. RT-PCR was performed using a Biorad system (Bio-Rad Laboratories, USA) with SYBR Green. Each PCR was performed in triplicate to a final solution volume of 20:10 μl of SuperReal PreMix Plus, 1 μl of diluted cDNA products, 0.6 μl of each paired primer, and 7.8 μl of RNase-free water. Protocols were as follows: initial denaturation for 15 min at 95°C, followed by 40 cycles denaturation for 10 s at 95°C, and extension for 30 s at 60°C. Last cycle for dissociation of SYBR Green probe was 15 s at 95°C, 30 s at 56°C, and 15 s at 95°C. Primer pairs for quantitative real-time PCR were as follows: Iba1, 5′- CTTCAGCTCTAGATGGGTCTTGG−3′ (sense) and 5′- AAGAGAGGTTGGATGGGATCAAC−3′ (anti-sense),GAPDH, 5′- ACCAGCTTCCCATTCTCAGC−3′ (sense) and 5′- GAAGGTCGGTGTGAACGGAT-3′(anti-sense). The mRNA levels of the Iba1 were calibrated against GAPDH mRNA and the fold difference between groups was calculated by the 2^−ΔΔ*Ct*^ method (32).

#### Western blotting

The samples were mixed with RIPA and PMSF (Beyotime, China) and homogenized on ice. The dissolved proteins were centrifuged at 10,000 × g for 10 min at 4°C, and the supernatants were collected for further detection. The concentrations were determined using a BCA protein assay kit (Beyotime, China). Samples containing 30 μg protein were loaded on a polyacrylamide gel (5% stacking gel, 10% resolving gel), run at 80 mv for electrophoresis, and then electrophoretically transferred to PVDF membranes (Bio-Rad, USA) at 200 mA for 1.5 h. Membranes were blocked with 5% milk in TBST for 1 h and then incubated overnight with the primary antibody, NF-κB (1:2,000, ab32536, Abcam, USA). GAPDH (1:10,000, Beyotime, China) was used as an internal control. After three TBS washes, the membranes were incubated with a horseradish peroxidase-conjugated secondary antibody, sheep anti-rabbit IgG (1:8,000, Beyotime, China) for 1 h. After washing three times with TBST, the membranes were incubated with chemiluminescence substrates (Millipore Corp, USA) for 3 min and exposed to X-ray film. The gray value was quantified by ImageJ 1.50i software (NIH).

### Statistical analysis

Quantitative data were presented as the mean ± SEM. In most cases, two-way ANOVA was used for statistical analysis, and multiple comparisons of individual groups were performed using Fisher's LSD test. Differences were considered statistically significant if the *p* value was <0.05. For the MWM test, the average escape latency in the first 5 days of training among different groups was evaluated by three-way repeated-measures ANOVA.

## Results

### Effects of minocycline in the OF test

**Figure 1:**
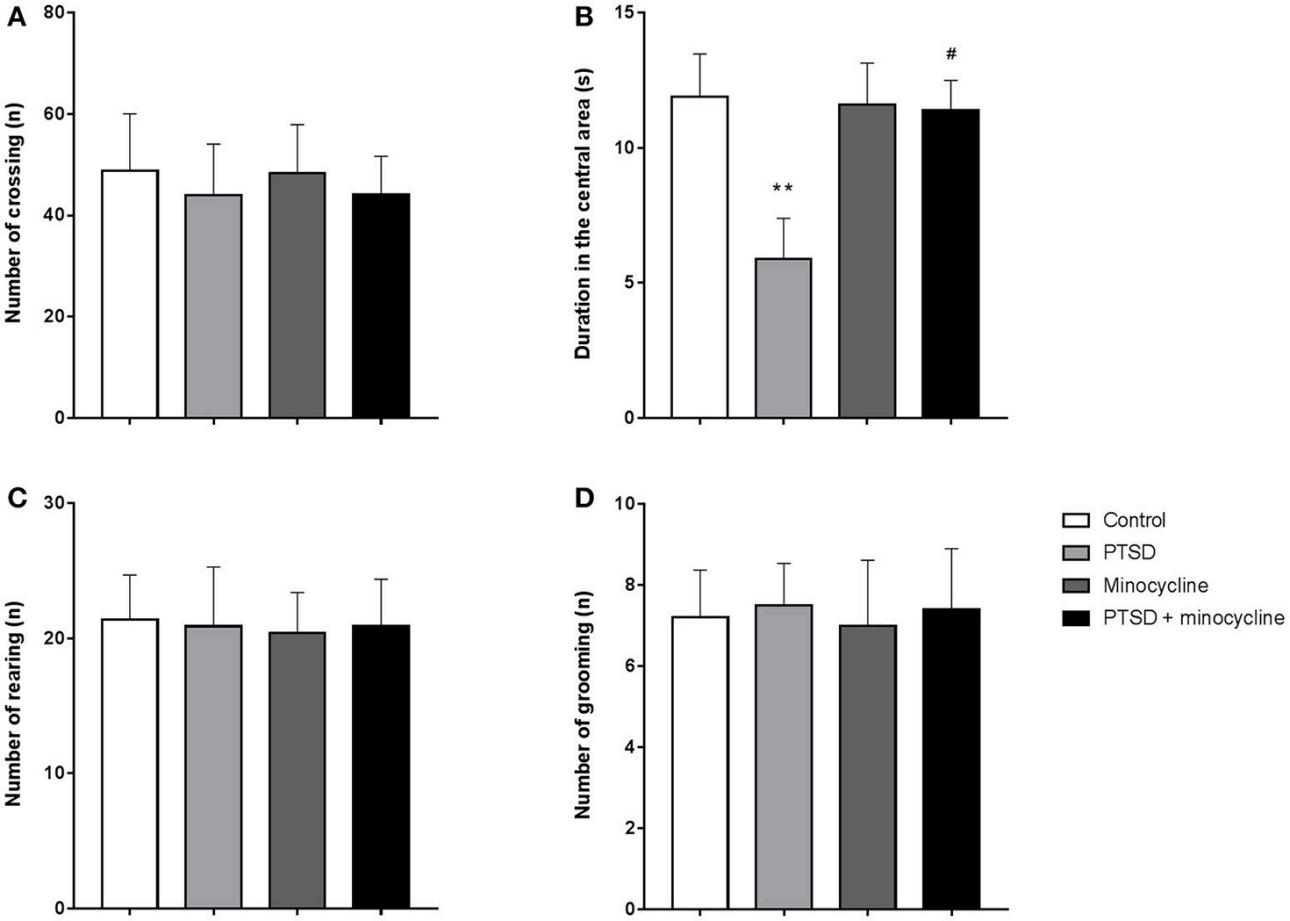
The effects of minocycline on the OF test. **(A)** The number of crossings in the open field. **(B)** Time spent in the central area of the apparatus. **(C)** The number of rearing events in the device. **(D)** The number of grooming events in the facility. The results are expressed as the mean ± SEM, *n* = 10, ^**^p < 0.01 vs. control; ^#^ *p* < 0.05 vs. PTSD.

For the duration of time spent in the central area, two-way ANOVA revealed a significant effect for IFS treatment [*F*_(1, 36)_ = 4.621, *p* < 0.05, Figure 1B], no effect was observed for minocycline-treatment and IFS-minocycline interaction. There was no effect was observed in the total number of crossing (Figure 1A), rearing (Figure 1C), and grooming (Figure 1D) in different groups. Fisher's LSD test confirmed that IFS-exposed rats showed a marked decrease in the time spent in the central area when compared with the control group (*p* < 0.01), and the central time of IFS-exposed rats treated with minocycline was significantly greater than that of IFS-exposed rats treated with vehicle (*p* < 0.05).

### Effects of minocycline in the EPM test

**Figure 2:**
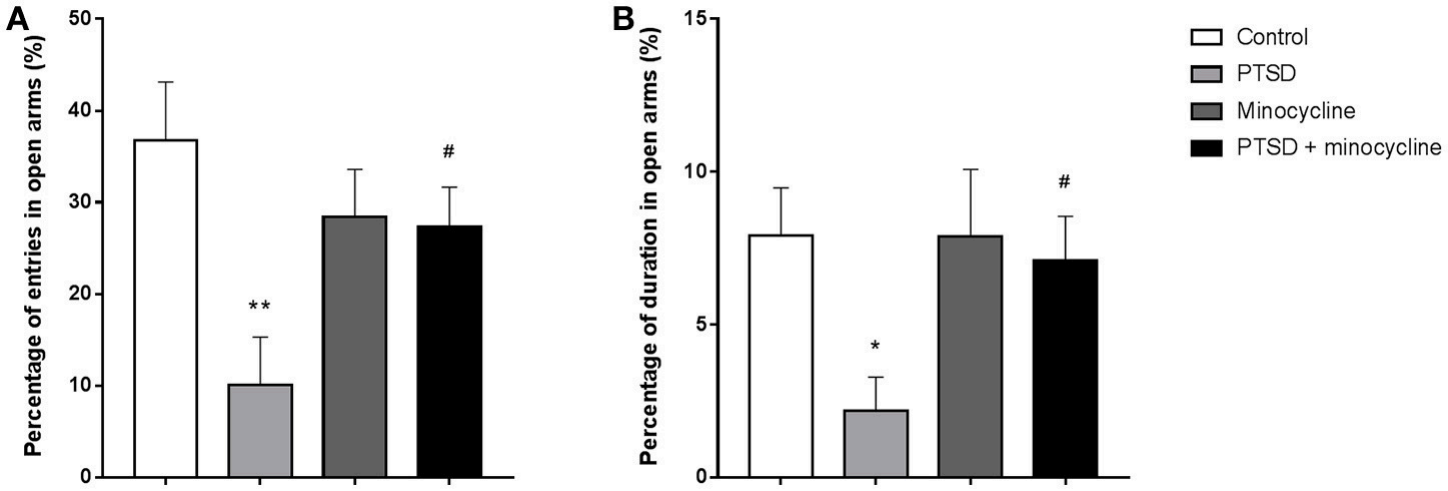
The effects of minocycline on the EPM test. **(A)** The percentage of entries in open arms. **(B)** The percentage of duration of time spent in open arms. The results are expressed as the mean ± SEM, n = 10, ^*^p < 0.05, ^**^p < 0.01 vs. control; ^#^ *p* < 0.05 vs. PTSD.

In terms of open-arm entries, two-way ANOVA revealed a significant effect for minocycline treatment [*F*_(1, 36)_ = 6.806, *p* < 0.05, Figure 2A] and an IFS-minocycline interaction [*F*_(1, 36)_ = 5.801, *p* < 0.05]. For the duration of time spent in open arms, there were no significant differences between groups (Figure 2B). Fisher's LSD test confirmed that IFS caused a significant reduction in the open arm entries (*p* < 0.01) compared with the control group. In addition, the IFS-exposed rats treated with minocycline exhibited a remarkable increase in the open-arm entries (*p* < 0.05) compared with the IFS-exposed rats treated with vehicle.

### Effects of minocycline in the MWM test

**Figure 3:**
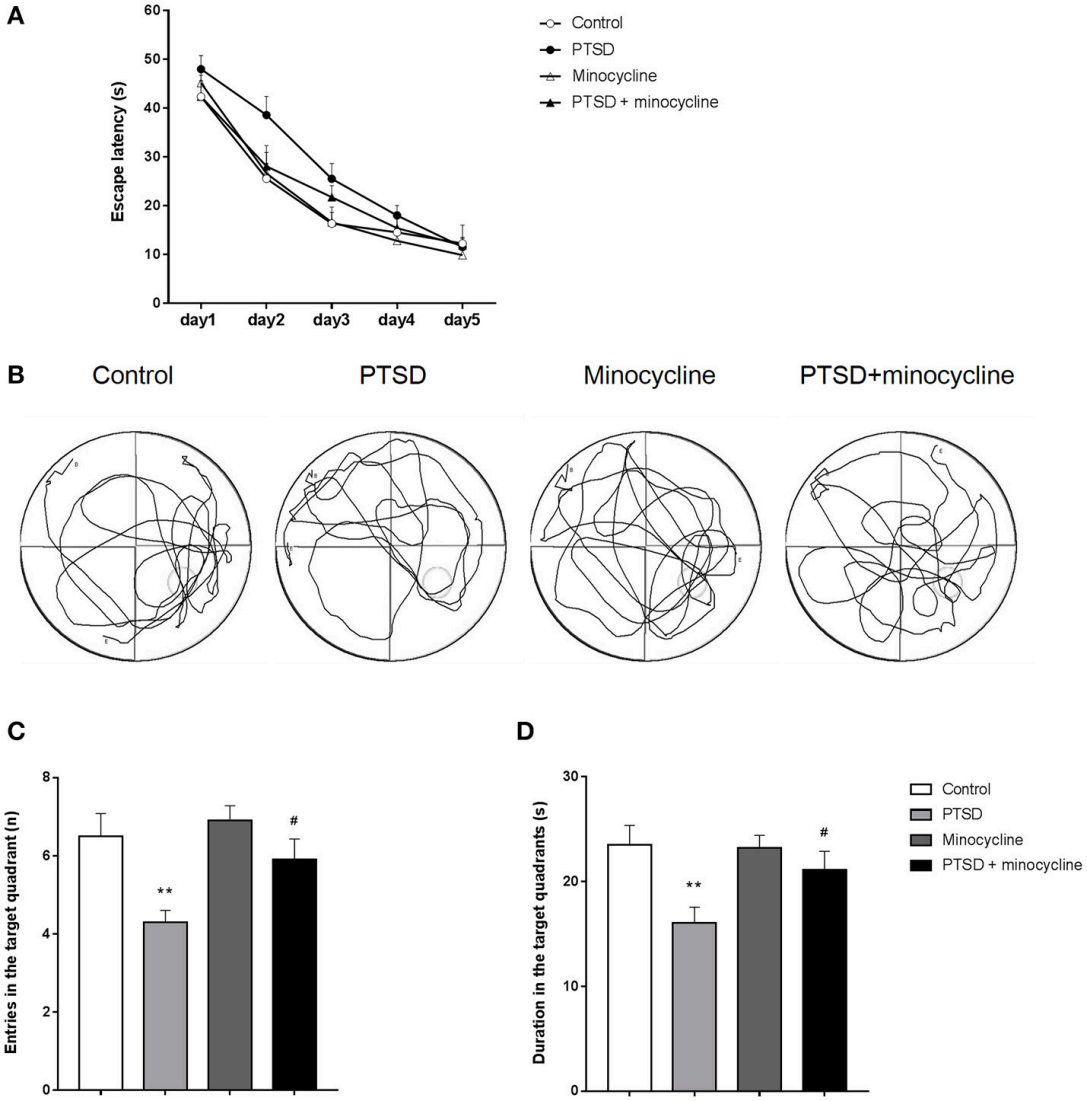
The effects of minocycline on the MWM test. **(A)** Average escape latency on the training day, taking into account the times of three sessions (excepted for the session starting in the target quadrant) per day. **(B)** One of the representative trajectories of the four groups in the water maze on the sixth day within 1 min (origination is opposite to the target quadrant). **(C)** Number of entries to the target quadrant on the sixth day of testing. **(D)** Duration of time spent in the target quadrant on the testing day. The results are expressed as the mean ± SEM, *n* = 10, ^**^p < 0.01 vs. control; ^#^ *p* < 0.05 vs. PTSD.

As shown in Figure 3A, three-way repeated-measures ANOVA revealed that escape latency of the four groups reduced over the 5 days training period [*F*_(4, 24)_ = 122.562, *p* < 0.001], and there was no interaction between days, IFS exposure and minocycline treatment [*F*_(4, 24)_ = 1.481, *p* > 0.05]. There was no IFS-minocycline interaction, day-IFS interaction and day-minocycline interaction [*F*_(1, 6)_ = 3.403, *p* > 0.05, *F*_(4, 24)_ = 0.593, *p* > 0.05, *F*_(4, 24)_ = 1.316, *p* > 0.05, respectively]. The IFS-exposed group had a longer escape latency during the training day on day 2 and day 3, while there was no significant difference between these groups.

As shown in Figure 3B, the IFS-exposed rats had fewer entries in the target quadrant compared with the control group, while treatment with minocycline increased the number of entries of the IFS-exposed rats compared to IFS-exposed rats treated with vehicle. In terms of entries in the target quadrant, two-way ANOVA revealed significant differences for IFS exposure [*F*_(1, 36)_ = 12.06, *p* < 0.01, Figure 3C] and minocycline treatment [*F*_(1, 36)_ = 4.712, *p* < 0.05]. In terms of duration in the target quadrant, two-way ANOVA revealed a significant difference for IFS exposure [*F*_(1, 36)_ = 8.892, *p* < 0.01, Figure 3D]. Fisher's LSD test confirmed that IFS-exposed rats showed a significant decrease in the number of entries (*p* < 0.01) and duration (*p* < 0.01) in the target quadrant compared with the control group, and administration of minocycline remarkably increased the number of entries (*p* < 0.05) and duration (*p* < 0.05) in the target quadrant compared with the vehicle treatment in IFS-exposed rats.

### Effects of minocycline on pro-inflammatory cytokines in the PFC and hippocampus

**Figure 4:**
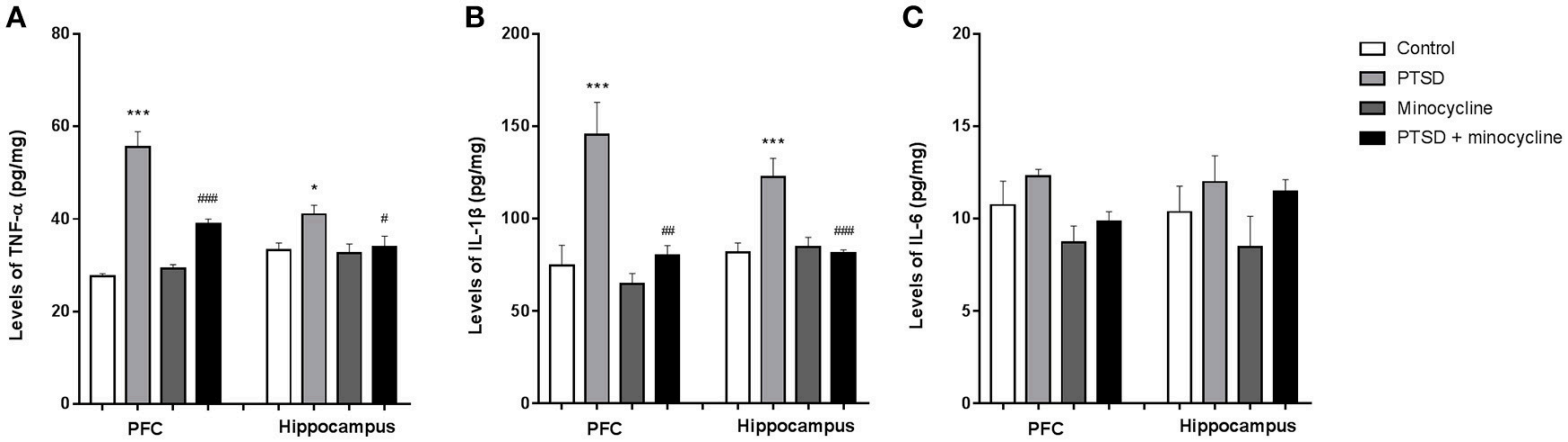
Effects of minocycline on pro-inflammatory cytokines in the PFC and hippocampus. **(A)** The levels of TNF-α in the PFC and hippocampus. **(B)** The levels of IL-1β in the PFC and hippocampus. **(C)** The levels of IL-6 in the PFC and hippocampus. The concentrations of cytokines in the tissue were detected by ELISA. The results are expressed as the mean ± SEM, *n* = 4, ^*^p < 0.05, ^***^p < 0.001 vs. control; ^#^ *p* < 0.05, ^##^ *p* < 0.01, ^###^ *p* < 0.001 vs. PTSD.

As shown in Figure 4A, in the levels of TNF-α in the PFC, two-way ANOVA revealed a significant effect for IFS exposure [*F*_(1, 12)_ = 105.9, *p* < 0.001], minocycline treatment [*F*_(1, 12)_ = 16.41, *p* < 0.01] and an IFS-minocycline interaction [*F*_(1, 12)_ = 24.59, *p* < 0.001]. In the levels of TNF-α in the hippocampus, two-way ANOVA revealed a significant effect for the IFS exposure [*F*_(1, 12)_ = 5.139, *p* < 0.05]. Fisher's LSD test confirmed that the IFS-exposed group showed a significant increase in the levels of TNF-α in the PFC (*p* < 0.001) and hippocampus (*p* < 0.05) compared with the control group. The levels of TNF-α in the PFC (*p* < 0.001) and hippocampus (*p* < 0.05) of IFS-exposed rats treated with minocycline were markedly lower than that of IFS-exposed vehicle treated rats.

As shown in Figure 4B, in the levels of IL-1β in the PFC and hippocampus, two-way ANOVA revealed a significant effect for IFS exposure [*F*_(1, 12)_ = 14.84, *p* < 0.01 and *F*_(1, 12)_ = 8.83, *p* < 0.05, respectively], a minocycline treatment effect [*F*_(1, 12)_ = 11.45, *p* < 0.01 and *F*_(1, 12)_ = 9.348, *p* < 0.01, respectively] and an IFS-minocycline interaction [*F*_(1, 12)_ = 6.158, *p* < 0.05 and *F*_(1, 12)_ = 12.47, *p* < 0.01, respectively]. Fisher's LSD test confirmed that IFS-exposed rats exhibited a significant increase in the levels of IL-1β in the PFC (*p* < 0.001) and hippocampus (*p* < 0.001) compared with control rats, and minocycline-treated IFS rats exhibited a significant decrease in the levels of IL-1β in the PFC (*p* < 0.01) and hippocampus (*p* < 0.001) compared with IFS-exposed rats treated with vehicle.

As shown in Figure 4C, in the levels of IL-6 in the PFC, two-way ANOVA revealed a significant difference for minocycline administration [*F*_(1, 12)_ = 6.874, *p* < 0.05]. In the levels of IL-6 in the hippocampus, no effect was observed between groups.

### Effects of minocycline on the activation of microglia in the PFC and hippocampus

**Figure 5:**
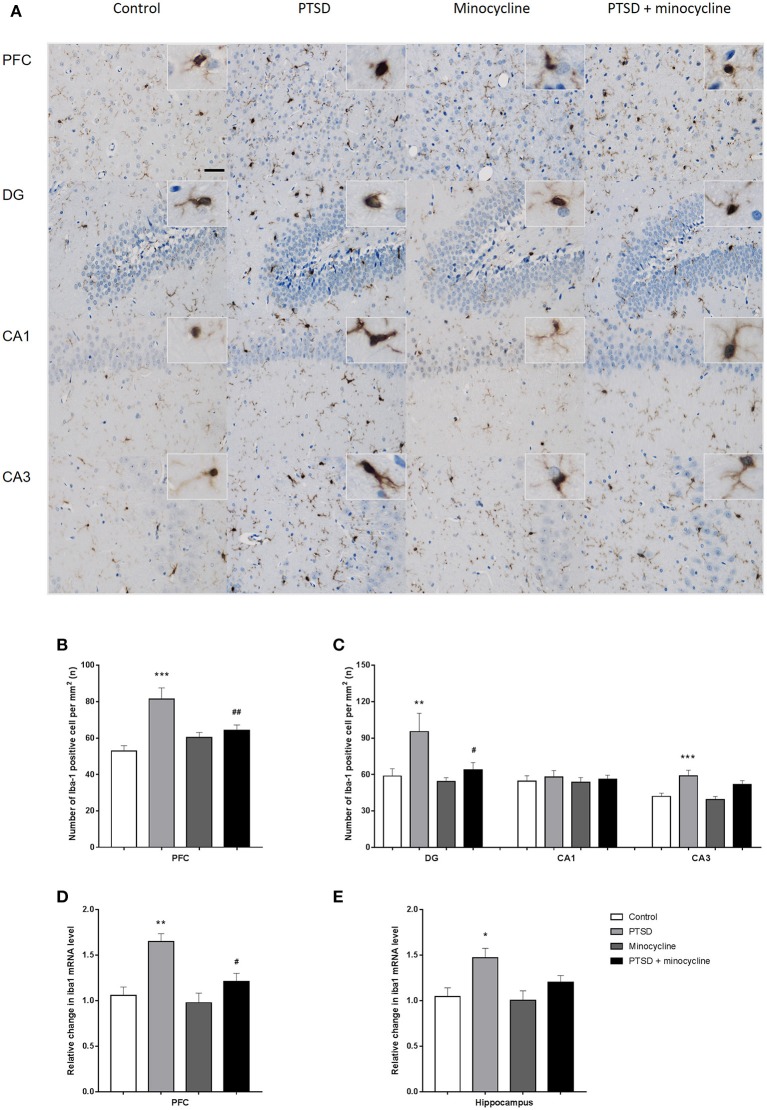
The effects of minocycline on microglial activation in the PFC and hippocampus. **(A)** One of the representative images of the PFC, DG, CA1, and CA3 of the hippocampus in the four groups. **(B)** The number of Iba-1-positive cells in the PFC. **(C)** The number of Iba-1-positive cells in the region of DG, CA1, and CA3 in the hippocampus. **(D)** Relative changes of the Iba1 mRNA levels in the PFC. **(E)** Relative changes of the Iba1 mRNA levels in the hippocampus. The results are expressed as the mean ± SEM. The number of Iba-1-positive cells was manually calculated from 10 sections of three rats of each group by ImageJ, and the relative levels of Iba1 mRNA were detected from three rats in different groups, ^*^p < 0.05, ^**^p < 0.01, ^***^p < 0.001 vs. control; ^#^*p* < 0.05, ^##^ *p* < 0.01 vs. PTSD. Scales bars = 50 μm.

In the number of microglia in the PFC, two-way ANOVA revealed a significant effect for IFS exposure [*F*_(1, 36)_ = 16.9, *p* < 0.001, Figure 5A] and an IFS-minocycline interaction [*F*_(1, 36)_ = 9.878, *p* < 0.01]. In the number of microglia in the DG, CA1 and CA3 of hippocampus, two-way ANOVA revealed a significant effect for IFS stress [*F*_(1, 36)_ = 6.861, *p* < 0.05, *F*_(1, 36)_ = 0.4354, *p* > 0.05, *F*_(1, 36)_ = 19.2, *p* < 0.001, respectively, Figure 5B] and a minocycline treatment effect [*F*_(1, 36)_ = 4.199, *p* < 0.05, *F*_(1, 36)_ = 0.08328, *p* > 0.05, *F*_(1, 36)_ = 2.232, *p* > 0.05, respectively]. Fisher's LSD test confirmed that IFS-exposed rats showed a significant increase in microglia number in the PFC (*p* < 0.001), DG (*p* < 0.01), and CA3 (*p* < 0.001) compared with the control rats, and the microglia number in the PFC (*p* < 0.01) and DG (*p* < 0.05) of minocycline-treated IFS-exposed rats was remarkably less than that of IFS-exposed vehicle-treated rats.

For the relative level of mRNA of Iba1 in the PFC, two-way ANOVA showed a significant effect for IFS exposure [*F*_(1, 8)_ = 19.61, *p* < 0.01, Figure 5D] and a minocycline treatment effect [*F*_(1, 8)_ = 7.598, *p* < 0.05]. For the relative level of mRNA of Iba1 in the hippocampus, two-way ANOVA showed a significant effect for IFS exposure [*F*_(1, 8)_ = 10.93, *p* < 0.05, Figure 5E]. Fisher's LSD test confirmed that IFS-exposed rats exhibited a significant increase in the relative level of mRNA of Iba1 in the PFC (*p* < 0.01) and hippocampus (*p* < 0.05) compared with the control rats, and the relative level of mRNA of Iba1 in the PFC (*p* < 0.05) of minocycline-treated IFS-exposed rats was significantly less than that of IFS-exposed vehicle-treated rats.

### Effects of minocycline on NF-κB in the PFC and hippocampus

**Figure 6:**
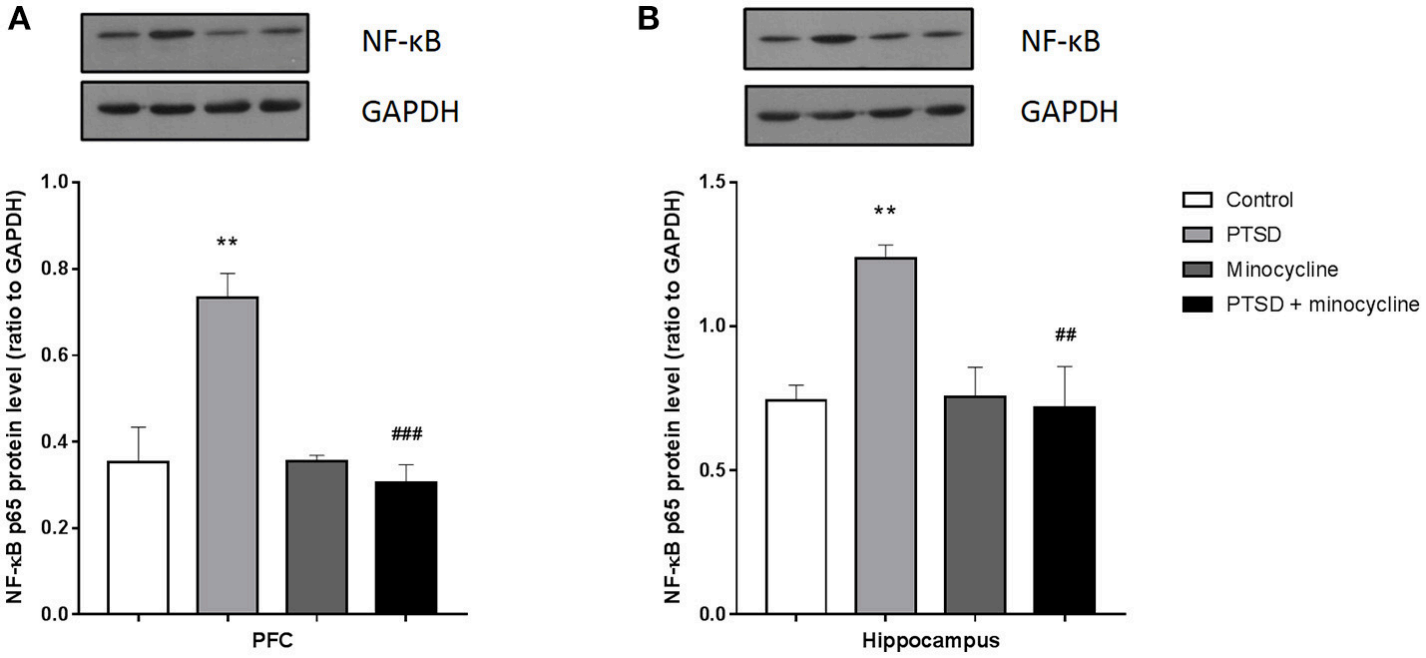
The effect of minocycline on the expression of NF-κB in the PFC and hippocampus. **(A)** The levels of p65 in the PFC. **(B)** The levels of p65 in the hippocampus. The protein levels were detected by western blotting, and each value was normalized to GAPDH. The results are expressed as the mean ± SEM, and the blot shown is one of 3 independent experiments, ^**^p < 0.01 vs. control; ^##^*p* < 0.01, ^###^*p* < 0.001 vs. PTSD.

For the levels of p65 in the PFC and hippocampus, two-way ANOVA showed a significant effect for IFS exposure [*F*_(1, 8)_ = 9.398, *p* < 0.05, Figure 6A and F _(1, 8)_ = 5.877, *p* < 0.05, Figure 6B, respectively], a minocycline treatment effect [*F*_(1, 8)_ = 15.73, *p* < 0.01 and *F*_(1, 8)_ = 7.192, *p* < 0.05, respectively] and an IFS-minocycline interaction [*F*_(1, 8)_ = 16.04, *p* < 0.01 and *F*_(1, 8)_ = 8.038, *p* < 0.05, respectively]. Fisher's LSD test confirmed that the IFS-exposed rats showed a significant increase in the levels of p65 in the PFC (*p* < 0.01) and hippocampus (*p* < 0.01) compared with the control group, and minocycline significantly reduced the levels of p65 in the PFC (*p* < 0.01) and hippocampus (*p* < 0.01) compared to vehicle treatment in IFS-exposed rats.

## Discussion

The present study demonstrated that the 6 days IFS protocol was able to induce PTSD-like behavior, as evidenced by anxiety-like behavior in the OF test and the EPM test, as well as impairment of learning and memory in the MWM test. The behavioral changes were accompanied by a significant increase in the production of pro-inflammatory cytokines and activation of microglia and NF-κB in the PFC and hippocampus in the stressed animals. However, treatment with minocycline was able to reverse these behavioral alterations, and the neuroprotective effects of minocycline might be exerted through its anti-inflammatory properties via the NF-κB signaling pathway.

In the present study, we examined the effects of minocycline on the anxiety-like behaviors and learning and memory functioning in a rat model of PTSD induced by IFS. The OF test is designed to measure spontaneous activity and anxiety-like behaviors. The results of the OF test revealed that IFS induced anxiety-like behavior and did not affect spontaneous movement, while minocycline was able to attenuate anxiety-like behaviors, as indicated by the increased time spent in the central area. These results suggested that minocycline has an anxiolytic effect in a model of IFS, which is consistent with previous studies (22, 23, 33). The EPM is widely used to (22, 23, 33). The EPM is widely used to measure anxiety-like behavior in rodents, and it was validated pharmacological agent. Utilizing the IFS model of PTSD, administration of minocycline significantly reduced the IFS-induced anxiety-like behaviors, as evidenced by the increase in number of entries and duration of time spent in the open arms of the EPM. The MWM test assesses spatial learning and memory function in rodents. Our results showed that IFS caused impairment in spatial learning and memory, as shown by an increased latency in reaching the platform and reduced duration of time and frequency of entry in the target quadrant, which were reversed by the treatment with minocycline. These results were consistent with previous studies in rodents suggesting that with previous studies in rodents suggesting that minocycline is beneficial for cognitive functions Anxiety and cognitive impairment are common symptoms of PTSD and other psychiatric disorders, especially stressor-related disorders. However, the underlying mechanism of PTSD is not well understood. Our previous studies and other researchers noted that the hypothalamic-pituitary-adrenocortical (HPA) axis was involved in the response to stress, which manifested by enhanced negative feedback inhibition of the HPA axis (34) and excessive expression of corticotrophin-releasing factor receptor in the hypothalamus, amygdala and PFC (35). Furthermore, other studies have indicated that inflammation is a crucial mediator of the responses to various stressors and may lead to anxiety-like behavior and cognitive impairments. Previous studies have found that pro-inflammatory cytokines were increased in rodent models of PTSD and patients suffering from PTSD. For example, studies on rodent models of PTSD indicated higher levels of the pro-inflammatory cytokines in the hippocampus and PFC (14, 36, 37). A meta-analysis reported that the levels of IL-6, IL-1β, TNF-α and interferon-γ were up-regulated in patients with PTSD compared with controls (38). Our results also indicated the protein levels of TNF-α, IL-1β, and IL-6 were increased in the PFC and hippocampus. The microglia act as the resident macrophages in the brain and produce pro/anti-inflammatory cytokines. We also examined the relative levels of mRNA and protein of Iba-1, a marker of microglia, in the PFC and hippocampus. The qPCR and IHC results indicated that IFS increased the level of mRNA of iba-1, the number of Iba-1-positive cells and changed the morphology of the microglia from hyper-ramified to amoeboid shape in the PFC and hippocampus. Consistent with our results, Sun et al. also found up-regulation of Iba-1 in the CA1 and CA3 regions of the hippocampus in the animal model of PTSD induced by single prolonged stress (36). Similarly, other studies also reported that various stressors could induce microglial activation in different animal models (39–42). These increased microglial responses are likely part of the neuroinflammatory responses in which microglial activation is the major cellular response to CNS dysfunction. The presence of morphological and immunological findings suggests a potential role for microglia and neuroinflammation in the pathogenesis of the PTSD. It is well known that NF-κB is a crucial regulator of immunological processes. O'Donovan et al. found an up-regulation of target genes of NF-κB in patients suffering from PTSD compared with non-PTSD controls (43). Pace et al. analyzed the circulating peripheral mononuclear cells of child abuse victims with PTSD and reported greater NF-κB signaling activity in female childhood abuse victims with PTSD than non-PTSD controls (44). In addition, a model of PTSD induced by predator scent stress was found to be associated with overexpression of NF-κB in the hippocampus (45). Our results also suggested that NF-κB was activated in the brains of rats subjected to IFS. Pyrrolidine dithiocarbamate, an inhibitor of NF-κB, was found to normalize anxiety-like behavior, as well as startle response and startle habituation in rodents (45). Si et al. indicated that NF-κB activity in the basolateral amygdala was essential for memory reconsolidation and may be a potential target for pharmacological treatment for PTSD (46). Since the NF-κB is a critical factor in neuroinflammation, an inhibitor of NF-κB signaling might attenuate PTSD symptoms by down-regulating inflammation.

The choices for pharmacological treatment for PTSD are limited, although selective serotonin reuptake inhibitors (SSRI) are the mainstay treatment. In addition, aspirin and brufen were used in clinical trials to reduce neuroinflammation in patients suffering from PTSD (5). Our findings demonstrated that administration of minocycline significantly reduced the levels of pro-inflammatory cytokines and the activation of microglia and NF-κB in a rat model of PTSD induced by IFS. Previous studies have reported that a single dose of minocycline had a potential effect of preventing the deterioration of behavior and exaggeration of neuroinflammation (14). Similarly, a longer injection of minocycline also attenuated single prolonged stress-induced anxiety-like behavior (36). In addition, minocycline was able to improve cognitive deficits in a cerebral microvascular amyloid model (47). Minocycline has been found to inhibit the activation of microglia *in vivo* (26). Moreover, researchers have suggested that minocycline can selectively inhibit microglia polarization to the pro-inflammatory state (27). Furthermore, previous studies indicated that minocycline affected the mRNA and protein expression of NF-κB in microglia (27). In the present study, our results suggested that treatment with minocycline was able to inhibit the NF-κB pathway, attenuate neuroinflammation and alleviate IFS-induced behavioral disturbances in rats.

In conclusion, our study demonstrated that a 6 days IFS protocol was able to induce PTSD-like behavior, elevate pro-inflammatory cytokines and activate the microglia and NF-κB in the PFC and hippocampus, which suggested that neuroinflammation is involved in the response to stress. Minocycline can attenuate these behavioral and molecular alterations, suggesting that minocycline might be a potential pharmacological agent for the treatment of PTSD. Although minocycline has been considered a safe antibiotic and anti-inflammatory agent for humans, further clinical trials are required to assess its therapeutic efficacy in clinical population with PTSD.

## Author contributions

WW performed the major experiments, data analysis, and wrote the manuscripts. RW, JX, XQ, HJ, and AK established the biophysical model, DL and FP designed this study. DL, CH, and RH revised the paper.

### Conflict of interest statement

The authors declare that the research was conducted in the absence of any commercial or financial relationships that could be construed as a potential conflict of interest.
